# Heterologous Biosynthesis of Five New Class II Bacteriocins From *Lactobacillus paracasei* CNCM I-5369 With Antagonistic Activity Against Pathogenic *Escherichia coli* Strains

**DOI:** 10.3389/fmicb.2020.01198

**Published:** 2020-06-19

**Authors:** Yanath Belguesmia, Kamel Bendjeddou, Isabelle Kempf, Rabah Boukherroub, Djamel Drider

**Affiliations:** ^1^UMR Transfrontalière BioEcoAgro N° 1158, Université de Lille, INRAE, Université de Liège, UPJV, YNCREA, Université d’Artois, Universite du Littoral Côte d’Opale, ICV – Institut Charles Viollette, Lille, France; ^2^Laboratoire de Microbiologie Appliquée, Faculté des Sciences de la Nature et de la Vie, Université de Bejaia, Béjaïa, Algeria; ^3^ANSES, Laboratoire de Ploufragan-Plouzané-Niort, Unité Mycoplasmologie Bactériologie Antibiorésistance, Ploufragan, France; ^4^Université Bretagne Loire, Rennes, France; ^5^Université de Lille, CNRS, Centrale Lille, ISEN, Univ. Valenciennes, UMR 8520 – IEMN, Lille, France

**Keywords:** multibacteriocinogenic strain, *Lactobacillus paracasei*, new class II bacteriocins, *Escherichia coli*, antimicrobials

## Abstract

*Lactobacillus paracasei* CNCM I-5369 isolated from a traditional Algerian dairy product produces extracellular inhibitory substances, namely, bacteriocins, which are active against a panel of pathogenic *Escherichia coli* strains. This activity was observed only at a narrow pH 4.5–5, and resulted to be heat stable and sensitive to the action of proteolytic enzymes, which indicate a proteinaceous nature. This new strain has a genome of 2,752,975 bp, with a 46.6% G + C ratio and contains at least 2664 coding sequences. The Bagel software analysis identified five open reading frames (ORFs) that are translated to new class II bacteriocin. Each ORF was cloned in frame with a His-tag tail and expressed in *E. coli* BL21 (DE3) (pLysS) strain. Of note, each fusion protein carrying any of these ORFs at the C- or N-terminal position resulted to be active against *E. coli* 184 strain used as target organism. This manuscript reports the first multi-bacteriocinogenic strain producing five new class II bacteriocins with activity against Gram-negative bacilli (GNB), namely, *E. coli*. Heterologous expression and activity of each new class II bacteriocin were demonstrated.

## Introduction

Bacteriocins are a family of ribosomally synthesized antimicrobial peptides (AMP) of low molecular sizes and proteinaceous nature. They are produced by Gram-negative bacteria (GNB) and Gram-positive bacteria (GPB) (Drider and Rebuffat, 2011). Bacteriocins from GPB are abundantly and diversely produced by lactic acid bacteria (LAB). They can be of narrow spectra, acting therefore only on members of the same species, or of broad spectra targeting other species and genera (Cotter et al., 2013). Bacteriocin classification is evolving with accumulated new knowledge, and there is not a single classification scheme that is universally accepted. Alvarez-Sieiro et al. (2016) classified bacteriocins into three classes. Briefly, class I contains small post-translationally modified peptides, designated RiPPs and are less than 10 kDa; class II contains unmodified bacteriocins smaller than 10 kDa; and class III contains unmodified bacteriocins larger than 10 kDa and endowed with bacteriolytic or non-lytic mechanisms (Alvarez-Sieiro et al., 2016). Unlike non-ribosomally synthesized peptides (NRPS), LAB-bacteriocins are synthesized and secreted during bacterial metabolism (Heng et al., 2007), following a scheme of primary metabolites (Lu et al., 2005). Moreover, LAB-bacteriocins were assumed to have limited toxicity for eukaryotic cells (Belguesmia et al., 2011; Dicks et al., 2018). LAB-bacteriocins have been used in the food sector to preserve foodstuffs from different bacterial contaminations attributable to spoilage and pathogens microorganisms. The last two decades have seen a stream of reports associating LAB-bacteriocins with a variety of multifaceted activities (Drider et al., 2016; Chikindas et al., 2018; Todorov et al., 2019). Bacteriocins represent an important alternative to traditional antibiotics in the face of antimicrobial resistance. Of note, LAB-bacteriocins can be active at nanomolar concentrations, unlike traditional antibiotics. They have two recognized modes of action (MoA). The first is associated with their capability to interact with the charged bacterial cell membrane, leading to ATP depletion, ionic imbalance, and membrane potential disruption (Naghmouchi et al., 2007). This MoA, known as a pore-forming mechanism, is mediated by positive charges of LAB-bacteriocins and the negatively charged bacterial membrane through electrostatic interactions (Egan et al., 2016; Vieco-Saiz et al., 2019). The second MoA is based on the use of a specific receptor located on the cell membrane. Nisin, which is a class I bacteriocin or lantibiotic, can target the lipid-anchored precursor of peptidoglycan (lipid II), which is a key component of peptidoglycan biosynthesis. This mechanism causes pore formation in the bacterial cell membrane, and consequently cell death (Bierbaum and Sahl, 2009; Egan et al., 2016; Vieco-Saiz et al., 2019). LAB-bacteriocin–lipid II interactions result in a loosened peptidoglycan meshwork, and ultimately in the leakage of vital cytosolic components by the formation of pores. Importantly, lipid II is also a target of the glycopeptide antibiotic vancomycin. Lipid II is not the unique receptor of LAB-bacteriocins; other ones have been described in the literature (Cotter et al., 2013; Cotter, 2014). Gram-negative pathogens have proven to be recalcitrant to LAB-bacteriocins by limiting access to the cell membrane. The discovery of LAB-bacteriocins with antimicrobial activity against GNB is of paramount interest for academic and applied research. Class II bacteriocins reported here constitute the first models to be simultaneously produced by a single LAB strain, and their activity against *Escherichia coli* carrying *mcr-1* gene opens a new window for further study.

In this paper, we report a multibacteriocinogenic strain, *Lactobacillus paracasei* CNCM I-5369, capable of producing five new class II bacteriocins with anti-*E. coli* activity. Each of these new class II bacteriocins was successfully cloned and heterologously expressed.

## Materials and Methods

### Bacterial Strains and Culture Conditions

Bacteria used in this work are listed in Table 1. All target *E. coli* strains were propagated, without agitation, in Brain Heart Infusion (BHI) media at 37°C for 12–18 h before use. *Lb. paracasei* CNCM I-5369 was grown at 37°C for 18–24 h, without agitation, in MRS medium (de Man, Rogosa and Sharpe) (De Man et al., 1960).

**TABLE 1:** Bacteria used in this study.

Strain	Source/References
**Producer strain**	
*Lactobacillus paracasei* CNCM I-5369	This work
**Heterologous producer strain**	
*Escherichia coli* BL21 (DE3) (pLysS)	Promega
**Target strains**	
*Escherichia coli*	ATCC 8739 Manassas, VA (USA)
*E. coli*	ATCC 25922 Manassas, VA (USA)
*E. coli*	CIP 7628 Paris (France)
*E. coli* 184*^#^	Al Atya et al., 2016
*E. coli* 289*	Al Atya et al., 2016
*E. coli* SBS 363	CIP Paris (France)
*E. coli* E4A4	ANSES Ploufragan (France)

ATCC, American Type Culture Collection; CIP, Collection Institut Pasteur. ANSES: French Agency for Food, Environmental and Occupational Health & Safety. *Resistant strain to colistin. ^#^Strain harboring the plasmid borne *mcr*-1 gene responsible for resistance to colistin.

### Bacteria Identification

The bacteriocinogenic strain used in this work was first identified as *Lb. paracasei* with classical methods of bacteriology based on different criteria such as the Gram staining, catalase activity, and sugar assimilation profile. This identification was confirmed with the MALDI-TOF Mass Spectrometry and 16S rRNA gene sequencing. The mass spectrometry (MS) profile was done as recently reported by Zidour et al. (2017). Genes encoding 16S rRNA were PCR-amplified from the total DNA extracted with the Wizard^®^ Genomic DNA Purification Kit (Promega, Charbonnières-les-Bains, France), and primers formerly designed by Drago et al. (2011) (Table 2).

**TABLE 2:** Oligonucleotide primers used in this study.

PCR	Primer design	Sequences
Amplification of 16S rRNA gene	16S Forward	5′-AGAGTTTGATCMTGGCTCAG-3′
	16S Reverse	5′-GGMTACCTTGTTACGAYTTC-3′
Check of recombinant plasmids	ORF010 Forward	5′-AGATCACTGGCGGTTTTGC-3′
	ORF 010 Reverse	5′-GCACCACCAGCAATTTCATCT-3′
	ORF 012 Forward	5′-AGCTTGGAAAAGATTGCTGGT-3′
	ORF 012 Reverse	5′-CAGACTACCAGTAAGCACGC-3′
	ORF 023 Forward	5′-ACGTAAATTCCTGACAATGCTGA-3′
	ORF 023 Reverse	5′-GCCCTGCCATCCCTTTAAAA-3′
	ORF 030 Forward	5′-TCCAACGATCAAAGCAGAGC-3′
	ORF 030 Reverse	5′-ACAGCCGTCACATAAGCCTT-3′
	ORF 038 Forward	5′-CGGCAGGTATTGGATCAGGA-3′
	ORF 038 Reverse	5′-ACCCAACCGCTCCTAAGTTT-3′

The 16S rRNA codifying gene was amplified with the following PCR program: 94°C/5 min, 29 cycles at 94°C/1 min, 55°C/1 min, and 72°C/1 min and finally 72°C/5 min. PCR products were purified with a PCR purification kit (Qiagen, Courtaboeuf, France), and sequenced by Eurofins Genomics (Munich, Germany). The obtained 16S rDNA nucleotide sequence was blasted with the BLASTn online software^1^.

### Production and Purification of Antibacterial Molecule(s) Produced by *Lb. paracasei* CNCM I-5369 Strain

The kinetics of production of inhibitory compounds was followed for 72 h. Thus, 100 ml of a sterile MRS medium was inoculated with 1 ml of an overnight culture of *Lb. paracasei* CNCM I-5369 grown at 37°C in MRS medium. The OD_600__nm_ was measured after 2, 4, 6, 8, 10, 26, 48, and 72 h of growth at 37°C. Samples of 1 ml each were taken and centrifuged (8000 *g*, 4°C, 10 min), and the resulting cell-free supernatant (CFS) was assessed for its antibacterial activity as previously reported (Batdorj et al., 2006). Briefly, each sample of CFS was serially diluted in the MRS medium using the following dilution ratios: 1/2, 1/4, 1/8, 1/16, 1/32, and 1/64. Then 10 μl of each diluted CFS was deposited on 1% (w/v) BHI medium agar inoculated with the indicator strain, *E. coli* ATCC 8739, or *E. coli* 184 carrying *mcr-1* gene. The plates were incubated at 4°C for 1 h and then at 37°C for 18 h. The antibacterial activity was expressed in arbitrary units/ml (AU/ml), which correspond to the reciprocal of the highest dilution (2*^*n*^*), resulting in the inhibition of the indicator strain. The total activity expressed in AU/ml corresponds to 2*^*n*^* × 100 μl/volume deposited (μl) (Batdorj et al., 2006). The concentration of proteins present in each CFS was determined with the BCA (bicinchoninic acid) Assay protein kit (Sigma-Aldrich, St Louis, MO, USA), as recommended by the supplier.

To purify the active compound(s), a culture of *Lb. paracasei* CNCM I-5369, grown in MRS medium for 24 h to 30 h at 37°C, was centrifuged (8000 *g*, 4°C, 10 min), and 40 ml of the CFS was loaded onto a reversed-phase C18 (Agilent, Santa Clara, CA, USA) cartridge. A washing step was done with 40 ml of 10% (v/v) acetonitrile, followed-up by an elution with 40 ml of 20% (v/v) acetonitrile solution. The active fraction of ca. 40 ml was dried using SpeedVac and resuspended in 4 ml of ultrapure water. The active fraction, designed E20, was stored at 4°C until use.

### Spectrum of Activity of E20 Fraction

The E20 fraction was tested against *E. coli* strains listed in Table 1. The antimicrobial activity was done using the agar diffusion test. Plates were flooded with a target bacterial suspension of ∼10^7^ CFU/ml and dried at room temperature (20°C) for 15 min. Then, wells of ∼6 mm diameter and 4 mm depth were made in 1% (w/v) BHI soft agar. Next, 50 μl of E20 fraction of a total protein concentration of 16 mg/ml (pH 4.5 or pH 7.0) was introduced in these wells. Notably, aliquots of 50 μl of MRS medium adjusted to the same pH were deposited and used as controls. Plates were pre-incubated for 2 h at 4°C to stop the growth of the target strain and allow inhibitory compounds present in the CFS to diffuse through agar. Afterward, plates were incubated for 24 h at 37°C, and the antimicrobial activity was evaluated by measuring the diameter of the inhibition zones formed around wells.

### Nature of Antimicrobial Activity of E20 Fraction

The E20 fraction was treated with different proteases, such as proteinase K, trypsin, papain, pepsin, and α-chemotrypsin, and also by other enzymes such as α-amylase and lipase (Sigma Aldrich) at a final concentration of 2 mg/ml. These treatments aimed at identifying the nature of the substance(s) responsible for activity. Briefly, enzymes were prepared in an adequate buffer at a concentration of 20 mg/ml. Then, 100 μl of each solution was added to 1 ml of E20 fraction and incubated for 2 h at 37°C. The residual antimicrobial activity was determined against *E. coli* ATCC 8739 used as a target strain (Batdorj et al., 2006).

The stability of this antimicrobial activity was tested at different temperatures and pH ranges. The E20 fraction was treated for 5 min at temperatures varying from 60 to 100°C, and then for 10–20 min at a temperature of 120°C.

For the pH stability, the E20 fraction was adjusted with 1 M of sterile NaOH or HCl to pH values ranging from 2 to 10. The E20 fraction was then left at room temperature for 2 h. Afterward, the residual activity was determined against *E. coli* ATCC 8739 as previously reported (Batdorj et al., 2006).

### *In silico* Analysis of the Genome of *Lb. paracasei* CNCM I-5369 Strain

The genome of *Lb. paracasei* CNCM I-5369 was sequenced using the Illumina MiSeq and HiSeq 2500 technology platforms and 2 × 250 bp paired-end reads (University of Liège, Belgium). The functional annotation of predicted genes was done with the RAST online server^2^, which predicted open reading frames (ORFs), and appropriate annotation according to the free SEED database (Overbeek et al., 2014).

To identify putative bacteriocins, the genome of *Lb. paracasei* CNCM I-5369 was analyzed with the Bagel 3 online software^3^, and the amino acid sequence translation was done with the Jpred software^4^.

### Heterologous Expression of Each ORF and Assessment of Its Antibacterial Activity

Each ORF coding for a putative bacteriocin was cloned under the control of the inducible T7 promoter, with the e-Zyvec technology^5^. Each ORF was cloned either upstream (N-terminal position) or downstream (C-terminal position) of the His-Tag (6 His) tail. This strategy enabled us to obtain 10 recombinant plasmids. Each recombinant plasmid was then introduced in *E. coli* BL21 (DE3)(plysS) competent cells. The transformed *E. coli* cells were regenerated in the SOC (Super Optimal broth with Catabolic repression) medium for 1 h at 37°C with shaking, at 160 rpm, and then selected on the Luria-Bertani (LB) agar medium supplemented with ampicillin (100 μg/ml) + chloramphenicol (30 μg/ml) (Sigma Aldrich). After overnight incubation at 37°C, the colonies were checked for plasmid carriage, targeting the appropriate ORF. This was done with PCR, using primers listed in Table 2, and the following PCR program: 94°C/3 min, 30 cycles at 94°C/1 min, 60°C/30 s, and 72°C/45 s, and finally 72°C/10 min.

For the heterologous expression assays, overnight cultures of *E. coli* strain BL21 (DE3) (pLysS), harboring recombinant plasmids were diluted to 1% (v/v) in LB medium + ampicillin (100 μg/ml) and chloramphenicol (30 μg/ml), and then grew aerobically at 37°C, until they had reached an OD_600__nm_ of 0.8. The gene expression was induced by adding the isopropyl-β-*d*-thiogalactopyranoside (IPTG) at 1 mM (Sigma-Aldrich). Bacteria were let to grow for five additional hours. Samples were harvested by centrifugation (8000 *g*, 10 min, 4°C), and the pellets were washed in a phosphate buffer solution (PBS) (pH 7.4), and resuspended in 10 mM imidazole (Sigma-Aldrich) and PBS buffer (pH 7.9). Then, they were sonicated five times, with a cycle of 2 min each, and cells were then lysed. The separation of the cytoplasmic soluble fraction (CSF) from the cytoplasmic insoluble fraction (CIF) and cell debris was done by centrifugation (14,000 *g*, 15 min, 4°C). The CSF was filtered (0.45-μm-pore-size filter) and loaded onto a 1-ml nickel His-Trap chelating column (Thermo Fisher Scientific, Waltham, MA, USA). The column was washed with 30 mM imidazole and PBS (pH 7.9). Peptides encoded by cloned ORFs with His-Tag were eluted with 2 ml of 250 mM imidazole and PBS (pH 7.9). After a desalting step with PD miditrap columns (GE Healthcare Life Science, Pollard, United Kingdom). The obtained solution was adjusted to pH 4.5 with the acetic acid, and its activity was evaluated against *E. coli* ATCC 8739 and *E. coli* 184 colistin-resistant strain.

## Results

The bacteriocin-producing strain described here was initially identified as a *Lactobacillus* species. The MALDI-TOF-MS and 16 rRNA sequencing enabled its identification as *Lb. paracasei* with a high degree of confidence. According to the NCBI database, the strains related were *Lb. paracasei* M0116 with 99.79% of identity (accession number EU780145.1), *Lb. paracasei* SK04B2 (accession number KJ764645.1), and *Lb. paracasei* Y132 (accession number MK774551.1) with 99.72% identity.

### Kinetics of Production of Inhibitory Compound(s)

**FIGURE 1:**
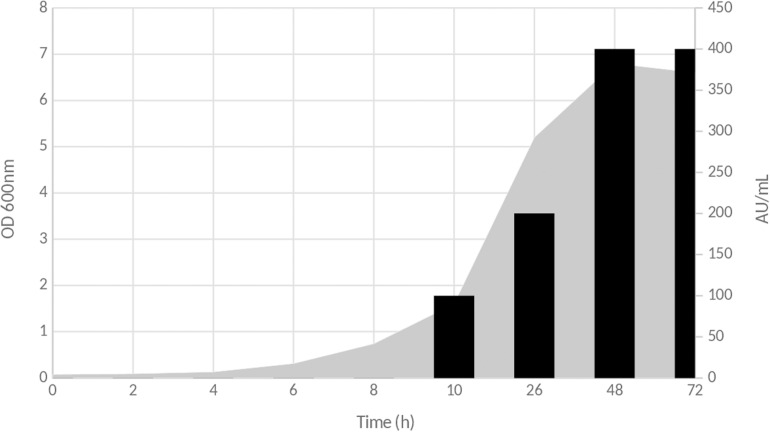
Time course production of inhibitory compound(s) during the growth of *Lb. paracasei* CNCM I-5369 on MRS medium. Gray zone corresponds to OD_600__nm_ evolution, and black bars represent antimicrobial activity in arbitrary units per milliliter (AU/ml).

The antimicrobial activity attributable to inhibitory compounds present in the CFS started to be detected after 10 h of culture on MRS medium at 37°C. This activity was estimated to 100 AU/ml and increased as seen in Figure 1. The highest activity was obtained at the end of the lag phase, with a total activity of 400 AU/ml (Figure 1). Then, it remained stable until the end of the experiment.

### The E20 Fraction Contains Putative Bacteriocin(s) With Activity Against *E. coli*

After each step of the purification procedure, several parameters including the antibacterial activity (AU/ml), total activity (UA), and specific activity (AU/mg) were determined. As reported in Table 3, the E20 fraction has a total activity of 8,000 AU.

**TABLE 3:** Antibacterial activities of the cell-free supernatant (CFS) and the semi-purified E20 fraction.

Fraction	Volume (ml)	Activity (AU/ml)	Total activity (UA)	Proteins (mg/ml)	Specific activity (AU/mg)
CFS	100	200	20,000	13.20	15.15
E20 fraction	20	400	8,000	15.98	25.03

The E20 fraction was adjusted to pH 4.5 and tested against different *E. coli* strains. Subsequently, all these target strains appeared to be, in a strain dependent-manner, sensitive to the action of E20 fraction. Notably, control assays were done at a same pH to discard any false-positive reaction attributable to the acidity of the medium (data not shown). Therefore, strong inhibition activities were observed on *E. coli* target strains based on the radii of inhibition zones formed around wells (Table 4).

**TABLE 4:** Cell-free supernatant (CFS) and E20 fraction antibacterial activities.

Strain	CFS	E20
*E. coli* ATCC8739	+	+ ⁣ +
*E. coli* ATCC25922	+	+ ⁣ +
*E coli* CIP7628	+	+ ⁣ +
*E. coli* 184	+	+ ⁣ +
*E. coli* 289	+	+ ⁣ +
*E. coli* SBS363	+	+ ⁣ +
*E. coli* E4A4	+	+ ⁣ +

*−: no activity; ±: 0 to 1 mm; +: 1 to 3 mm; ++: 3 to 6 mm. Interpretations done according to Batdorj et al. (2006).*

Notably, the activity of E20 fraction was lost following its treatment with proteolytic enzymes such as papain or proteinase K, but not with α-chymotrypsin or pepsin. These results indicate a proteinaceous nature of these antimicrobial substances. Notably, activity of E20 fraction remained stable for 20 min at 100°C but not for a longer heating period of incubation. Indeed, this antimicrobial activity decreased significantly after 30 min, at 100°C, and was completely abolished after 60 min, at the same temperature. Taken together, these results indicate that the E20 fraction might contain at least one active bacteriocin.

Additionally, the activity of E20 fraction was highly stable during storage at 4 to 8°C, with a very limited loss of its activity after 12 weeks. Importantly, the activity of E20 fraction was tightly pH-dependent. Indeed, at pH values ranging from 2 to 5, the E20 fraction showed a strong antimicrobial activity and then started to decrease drastically until disappearing at a pH of 6 or higher.

### Identification of Bacteriocin-Coding ORFs

**FIGURE 2:**
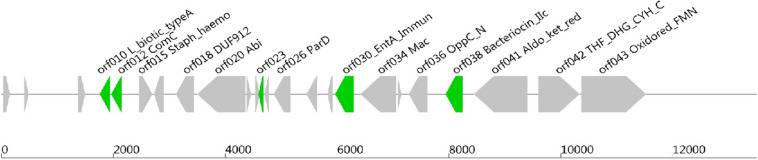
Open reading frames coding (ORFs), for presumed new class II bacteriocins, located on the chromosome of *Lb. paracasei* CNCM I-5369.

Software such as Bagel or AntiSmash are routinely used to identify genes coding for bacteriocins (van Heel et al., 2013; Weber et al., 2015; Collins et al., 2018). They have algorithms with a capability to recognize specific amino acids on the bacteriocin sequence, and the Bagel software is currently the most suitable one for this application (Oliveira et al., 2017; Collins et al., 2018). The genome of *Lb. paracasei* CNCM I-5369 contains 2,752,975 bp, with a 46.6% G + C ratio, and 2,664 coding sequences. The *in silico* analysis of this genome with the Bagel 3 tool^6^ enabled us to identify five ORFs (Figure 2) and reliably predict the likelihood that these sequences are translated to produce new class II bacteriocins with molecular sizes varying from 3.199 to 12.252 Da (Table 5). The first bacteriocin (ORF010) has a predicted molecular size of 3.199 Da and a pI of 5.17. Those of the ORF012 and ORF023 were 6.300 and 6.582 Da, with pI values of 4.86 and 8.25, respectively. The fourth and fifth ones (ORF030 and ORF038) have the largest molecular sizes with 10.395 and 12.252 Da, and pI of 8.62 and 6, respectively.

**TABLE 5:** Amino acid sequence of each putative new class II bacteriocins produced by *Lb. paracasei* CNCM I-5369.

Class	ORF number	Putative peptide sequence	Predicted masses (Da)
Class II	ORF010	MYTMTNLKDKELSQITGGFAFVIPVAAILGF LASDAWSHADEIAGGATSGWSLADKSHSL	6.300
Class II	ORF012	MQQFMTLDNSSLEKIAGGENGGL WSIIGFGLGFSARSVLTGSLF VPSRGPVIDLVKQLTPKN	6.582
Class II	ORF023	MLILGLIAIDAWSHTDQIIAGFLKGWQGM	3.199
Class II	ORF030	MTDKRETLMSMLSKAYANPTIKAEPAL RALIETNAKKVDEGDDEKAYVTAVTQL SHDISKYYLIHHAVPEELVAVFNYIKKDV PAADIDAARYRAQALAAGLVAIPIVWGH	12.252
Class II	ORF038	MYVKDSKVDLTQNNLLPFEEKRKIM SYNYRQLDDFQLSGVSGGKKKFDCA ATFVTGITAGIGSGTITGLAGGPFGIIGGA VVGGNLGAVGSAIKCLGDGMQ	10.395

**FIGURE 3:**
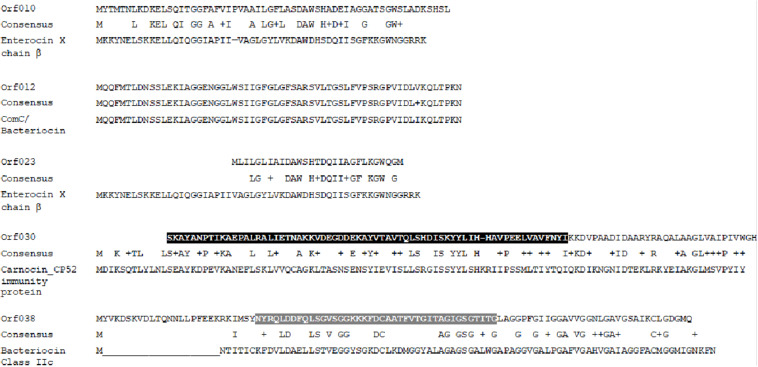
Sequence alignments of putative new bacteriocins translated from ORFs located and identified using the Bagel 3 software and the NCBI alignment tool (black zone = consensus domain of Ent_A Immun Superfamily; gray zone = conserved domain of class IIc bacteriocin).

Amino acid sequences identified with Bagel 3 and Blastp online software^1^ revealed some similarities between the bacteriocin predicted from ORF010 and enterocin X or Lactococcin-like family Lantibiotic (Hu et al., 2010). Of note, these new bacteriocins have only 24 common amino acids with the β-chain of Enterocin-X (Figure 3). The ORF012 may be translated into a bacteriocin similar to the ComC/BlpC leader-containing pheromone/bacteriocin family (Wang et al., 2018), while ORF023 matches with bacteriocins of the lactobin A/cerein 7B family (Escamilla-Martínez et al., 2017). The bacteriocin predicted from the ORF030 is 33% similar with carnobacteriocin B2 and a class IIa immunity protein (Quadri et al., 1995). Finally, the sequence predicted from ORF038 is 36% similar to that of thermophilin A, a bacteriocin with characteristics of class IIc (Marciset et al., 1997).

### Heterologous Expression and Activity of Each New Bacteriocin

**FIGURE 4:**
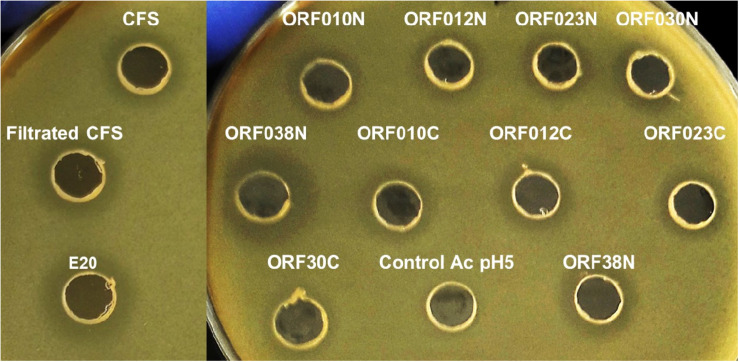
Agar diffusion test on *E. coli* 184 (*mcr-1*+) strain of (Control Ac pH5) PBS adjusted to pH 5 with acetic acid (negative-control), CFS (cell free culture supernatant); filtrated CFS (0.2 μM); E20 semi-purified fraction; N-terminal Histidine-tagged recombinant peptides (ORF010N, ORF012N, ORF023N, ORF030N, and ORF038N) and C-terminal Histidine-tagged recombinant peptides (ORF010C, ORF012C, ORF023C, ORF030C, and ORF038C).

Data depicted in Figure 4 and Table 6 report the activity of each new bacteriocin following its heterologous expression in *E. coli* BL21 (DE3) (pLysS). Remarkably, this activity was found for each ORF cloned in frame with a His-tag at the N- or C-terminal position. These activities obtained in a heterologous system were slightly different from those attributed to the native system, measured in the CFS or in the E20 fraction (Figure 4 and Table 3). Antimicrobial activities comprised between 200 and 800 AU/ml, and the upmost one was registered for ORF038, when its corresponding gene was cloned at the C-terminal position (His-tag at the N-terminal position). This activity was two to four times higher than those obtained from other constructions (Table 6).

**TABLE 6:** Antimicrobial activity of new class II bacteriocins as fusion-proteins against *E. coli* 184.

Recombinant peptide (N-terminal His-Tag)	Antimicrobial activity (AU/ml)	Recombinant peptide (C-terminal His-Tag)	Antimicrobial activity (AU/ml)
ORF010 HisTag N-ter	400	ORF010 HisTag C-ter	200
ORF012 HisTag N-ter	400	ORF012 HisTag C-ter	200
ORF023 HisTag N-ter	400	ORF023 HisTag C-ter	200
ORF030 HisTag N-ter	400	ORF030 HisTag C-ter	200
ORF038 HisTag N-ter	800	ORF038 HisTag C-ter	400

## Discussion

Bacteriocins are presently of common use in the food industry, in spite of their great potential for animal feeds, organic fertilizers, environmental protection, microbiota regulators, and personal care products. Nisin is the only LAB-bacteriocin to be approved by the FDA as a food preservative (E234) under the Annex of the EC regulation 1333/2008. Nevertheless, during the last decade, ca. 37% of published research on bacteriocins has focused on their potential as therapeutics (López-Cuellar et al., 2016; Mathur et al., 2017; Meade et al., 2020). LAB-bacteriocins differ in many aspects from traditional antibiotics (Cleveland et al., 2001; Perez et al., 2014), and their gene-encoded nature renders them genetically amendable and their activity improvable (Field et al., 2015).

By 2050, the expected number of deaths from antimicrobial resistance will increase to 10 million deaths per year globally (O’Neill, 2016), indicating that action to avert this crisis is needed. Major objectives should include new antibiotic discovery to rekindle the pipeline and the pursuit of antibiotic alternatives such as antimicrobial peptides. LAB-bacteriocins, which warrant serious consideration, are most often active against genetically related strains, and seldom against phylogenetically distant target strains such as GNB. LAB-bacteriocins having activity against GNB activity are of academic interest and will open new opportunities for medical applications, mainly to treat GNB-associated infections. The first anti-GNB LAB-bacteriocin was reported by Line et al. (2008) who reported the *in vitro* effectiveness of Enterocin E-760 against *Campylobacter jejuni*, a leading food-borne and human pathogen. Subsequently, Svetoch et al. (2011) reported the activity of a class IIa bacteriocin, named L-1077, against different *Salmonella* Typhimurium, *Salmonella* Enteritidis, and *E. coli*. Then, Messaoudi et al. (2012) reported the activity of the bacteriocin SMXD51 against *C. jejuni*. All these examples showed that activity of LAB-bacteriocins is not restricted to GPB, but can be active against GNB.

In this work, we report a *Lb. paracasei* CNCM I-5369 strain, capable of producing five new class II bacteriocins. The genome sequencing and analysis enabled us to locate at least five different ORFs coding for presumed new class II bacteriocins. Attempts to identify these new bacteriocins were first undertaken by the mass spectrometry approach on the CFS prepared from *Lb. paracasei* CNCM I-5369 culture and then on the semi-purified E20 fraction. Because of a very limited amount of pure peptides obtained at the end of the purification procedure, and because of their possible post-translational modifications, we decided to identify these new bacteriocins with a bioinformatics approach. Bacteriocins translated from ORF010 and ORF023 were less than 10 kDa and exhibited similarity with the β-chain of enterocin X, a class IIb bacteriocin (Hu et al., 2010). Interestingly, the GxxxG motif present in the amino acid sequence of bacteriocins encoded by ORF010 and ORF023 have been shown to play a role in the *sec*-independent export machinery of enterocin X (Nissen-Meyer et al., 2010; Escamilla-Martínez et al., 2017). Of note, ORF010 and ORF012 were separated only by 28 nucleotides, suggesting that their bacteriocins can be affiliated to class IIb. The BLASTP 2.9.0 software (Altschul et al., 1997) analysis done on sequences translated from ORF010 and ORF012 revealed large amino acid differences between them. Moreover, the predicted bacteriocin from ORF012 is highly similar to bacteriocins from the ComC/BlpC family (Hu et al., 2010). The bacteriocin predicted from ORF030 contains amino acid sequences partly similar to immunity proteins of Enterocin A, a class IIa bacteriocin organized in antiparallel form, with four alpha-helical globular bundles and a flexible fifth divergent C-terminal helical hairpin (Fimland et al., 2002). Finally, the bacteriocin predicted from ORF038 was associated with the thermophilin A bacteriocin and contains amino acids typical for class IIc bacteriocins (Marciset et al., 1997). Moreover, a clear ribosome binding site (RBS) was identified and located upstream of each located ORF (data not shown). Of note, the CFS and semi-purified E20 fraction prepared from *Lb. paracasei* CNCM I-5369 inhibited *E. coli* 184, a strain from swine origin carrying on a plasmid, the gene *mcr-1* gene, which is responsible for resistance to colistin. The discovery of plasmid-borne *mcr-1* gene was reported for the first time by Liu et al. (2016), and its rapid dissemination worldwide has consequently limited colistin as a drug of last resort for treatments of infections associated with multidrug-resistant GNB (Teo et al., 2016). Similarly, CFS and E20 fraction inhibited the growth of *E. coli* 289 strain from swine origin, which is as well resistant to colistin, but by a mechanism other than *mcr-1* gene. Of note, the activity obtained against the short-chain LPS *E. coli* SBS363 and the sensitive *E. coli* E4A4 strain were, surprisingly, in the same range as those obtained for other *E. coli* strains tested here.

As effective antimicrobials, LAB-bacteriocins may replace or prolong the effectiveness of antibiotics such as colistin (Naghmouchi et al., 2013). Here, we report a multibacteriocinogenic strain, *Lb. paracasei* CNCM I-5369, capable of inhibiting different *E. coli* strains including those resistant to colistin, through production of new class II bacteriocins. Noteworthy, each of these peptides was heterologously produced and its activity was confirmed on *E. coli* 184 strain. The activity obtained with E20 fraction may result from a synergistic interaction between these five new bacteriocins, in acidic pH, as it has been previously suggested for other bacteriocins (Yang and Ray, 1994; Houlihan et al., 2004).

In summary, we report a multibacteriocinogenic strain, namely, *Lb. paracasei* CNCM I-5369, with activity against *E. coli*. Analysis of the whole-genome sequences enabled us to identify ORFs presumed to code for five new class II bacteriocins. Each ORF was heterologously expressed in *E. coli* BL21 (DE3) (pLysS), and its activity was determined against *E. coli* 184 (*mcr-1*+). Independently of its C or N orientation in the fusion protein, each ORF was shown to be active against the aforementioned target strain. Further experiments aimed at understanding the regulation and expression of each ORF in the natural host constitute our next focus.

## Data Availability Statement

The datasets generated for this study are available on request to the corresponding author.

## Author Contributions

The manuscript was written through contributions of all authors. All authors have given their approval to the final version of the manuscript.

## Conflict of Interest

The authors declare that the research was conducted in the absence of any commercial or financial relationships that could be construed as a potential conflict of interest.

## Footnotes

1 https://blast.ncbi.nlm.nih.gov/Blast.cgi

2 http://rast.nmpdr.org/

3 http://bagel4.molgenrug.nl

4 www.compbio.dundee.ac.uk/jpred/

5 https://www.e-zyvec.com/

6 http://bagel.molgenrug.nl//
